# Metabolomic analysis of methyl jasmonate treatment on phytocannabinoid production in *Cannabis sativa*

**DOI:** 10.3389/fpls.2023.1110144

**Published:** 2023-03-21

**Authors:** Matthew T. Welling, Myrna A. Deseo, Martin O’Brien, Jacob Clifton, Antony Bacic, Monika S. Doblin

**Affiliations:** Australian Research Council Research Hub for Medicinal Agriculture, La Trobe Institute for Sustainable Agriculture and Food, Department of Animal, Plant and Soil Sciences, School of Agriculture, Biomedicine and Environment, La Trobe University, Bundoora, VIC, Australia

**Keywords:** *Cannabis sativa*, phytocannabinoids, oxylipin, methyl jasmonate, jasmonic acid, liquid chromatography, mass spectrometry, untargeted metabolomics

## Abstract

*Cannabis sativa* is a multi-use and chemically complex plant which is utilized for food, fiber, and medicine. Plants produce a class of psychoactive and medicinally important specialized metabolites referred to as phytocannabinoids (PCs). The phytohormone methyl jasmonate (MeJA) is a naturally occurring methyl ester of jasmonic acid and a product of oxylipin biosynthesis which initiates and regulates the biosynthesis of a broad range of specialized metabolites across a number of diverse plant lineages. While the effects of exogenous MeJA application on PC production has been reported, treatments have been constrained to a narrow molar range and to the targeted analysis of a small number of compounds. Using high-resolution mass spectrometry with data-dependent acquisition, we examined the global metabolomic effects of MeJA in *C. sativa* to explore oxylipin-mediated regulation of PC biosynthesis and accumulation. A dose–response relationship was observed, with an almost two-fold increase in PC content found in inflorescences of female clones treated with 15 mM MeJA compared to the control group. Comparison of the inflorescence metabolome across MeJA treatments coupled with targeted transcript analysis was used to elucidate key regulatory components contributing to PC production and metabolism more broadly. Revealing these biological signatures improves our understanding of the role of the oxylipin pathway in *C. sativa* and provides putative molecular targets for the metabolic engineering and optimization of chemical phenotype for medicinal and industrial end-uses.

## 1 Introduction

*Cannabis sativa* L. is a predominantly dioecious plant species and is also one of the most widely used illicit drugs (Small and Cronquist, 1976; Peacock et al., 2018). Applications of this multi-use plant species include industrial (Karche, 2019), ornamental (Hesami et al., 2022), and pharmaceutical (Sharma et al., 2022). Domestication of *C. sativa* for food, fiber and medicine is thought to have spanned several millennia (Russo et al., 2008; Zhou et al., 2011). After a prolonged period of underutilization throughout much of the 20^th^ century, a number of licit large-scale multi-billion-dollar industries based around this plant are now emerging globally (Chandra et al., 2017). Over the last decade, the legitimacy of this plant as a *bona fide* medicine for pain alleviation and for the treatment of childhood epilepsy has been substantiated in a series of large-scale human clinical trials (Devinsky et al., 2016; Devinsky et al., 2017), resulting in the approval of the *C. sativa*-based medicine Epidiolex by central regulatory agencies such as the U.S. Food and Drug Administration (FDA), European Medicines Agency (EMA) and the Australian Therapeutic Goods Administration (TGA) (Chandra et al., 2017).

Biological activity of *C. sativa* in human therapy is attributed to the low molecular weight chemical constituents (specialized metabolites) it produces, with a class of isoprenylated resorcinyl polyketides referred to as phytocannabinoids (PCs) largely responsible for medicinal effects (Welling et al., 2022). While the role(s) of PCs within *C. sativa* has not been fully established, the concentration of these compounds surrounding the ovules of female flowers and on the anthers of male flowers suggests they may act as plant defense compounds against (a)biotic stressors (Ries et al., 2000; Sirikantaramas et al., 2005; Liu et al., 2019). Female plants are prolific producers of PCs, with capitate stalked trichomes on the vegetative tissues proximal to pistillate flowers being the major site for PC biosynthesis and accumulation (Livingston et al., 2020). Despite the inherent capacity of *C. sativa* plants to produce large quantities of PCs, the genetic and molecular components regulating PC content and accumulation within trichomes are only partially resolved (Grassa et al., 2021; Hesami et al., 2023). *C. sativa* is generally classified based on its primary agronomic purposes, with plants grown for industrial uses, such as for food, fiber, building material and seed, having strict limits on the concentration of the psychoactive intoxicant PC Δ9-tetrahydrocannabinol (Δ9-THC); generally, less than 0.5-1.0% depending upon the jurisdiction. Cannabidiol (CBD), considered to act as an analgesic, is the active pharmaceutical ingredient in Epidiolex. CBD along with Δ9-THC and their respective carboxylated precursors, cannabidiolic acid (CBDA) and Δ9-tetrahydrocannabinolic acid A (Δ9-THCA), are the most commonly occurring PCs among modern domesticated forms of *C. sativa* (Welling et al., 2020). However, a subset of the gene pool produces unusually high levels of CBD- and Δ9-THC-type analogues with shorter C_3_ alkyl side chains that are of increasing interest as potential modulators of the human endocannabinoid pathway (Izzo et al., 2009; Welling et al., 2019; Welling et al., 2020).

Understanding of PC biosynthesis, regulation, and its compartmentalization within trichomes has accelerated in recent years (Livingston et al., 2020), with many of the enzymes having been functionally validated (Hesami et al., 2020; Grassa et al., 2021). Biosynthesis begins in the cytosol with the activation of a fatty acid (FA) precursor by a acyl-activating enzyme (AAE) (Stout et al., 2012). Condensation of acyl-CoA with three molecules of malonyl-CoA by a cytosolic type III polyketide synthase (tetraketide synthase (TKS)) and a bacterial-like polyketide cyclase (olivetolic acid cyclase (OAC)) forms a resorcinyl intermediate (Taura et al., 2009; Gagne et al., 2012). This is then fused with the methylerythritol 4-phosphate (MEP) pathway intermediate, geranyl diphosphate (GPP), in the chloroplast by an aromatic prenyltransferase (PT) (Page and Boubakir, 2011). From the chloroplast, cannabigeroids with a linear isoprenoid moiety, such as cannabigerolic acid (CBGA), are predicted to be transported into the sub-cuticular storage cavity of glandular trichomes along with Δ9-THCA synthase (THCAS) and CBDA synthase (CBDAS). These covalently flavinylated synthases facilitate oxidative cyclization of the isopropyl moiety of CBGA, producing Δ9-THCA and CBDA. Lastly, these acidic PCs are non-enzymatic decarboxylated to form Δ9-THC and CBD (Sirikantaramas et al., 2004; Dussy et al., 2005; Taura et al., 2007). Like many type III polyketide synthases, TKS and its accessory protein OAC can accept various acyl-CoA starter units during polyketide assembly, and alkyl side chain substrate promiscuity has been demonstrated for the remaining steps of the pathway (Taura et al., 2009; Luo et al., 2019), with C_3_ alkyl side chain analogues of CBD (cannabidivarin (CBDV)) and Δ9-THC (Δ9-tetrahydrocannabivarin (THCV)) formed by butanoyl-CoA (Luo et al., 2019).

While many key steps in the PC pathway have been characterized, the biosynthetic and genetic origin of the acyl-CoA precursors which form the resorcinyl intermediate and direct alkyl side chain analogue composition remain unresolved. Several biosynthetic pathways are capable of contributing to the pool of acyl-CoA polyketide precursors (**Supplementary Figure S1**). For example, bitter acids, which are also prenylated phenolic compounds like PCs, are synthesized in the glandular trichomes of the closely related species *Humulus lupulus* (hops) by branched-chain amino acid (BCAA) precursors: leucine, valine, and isoleucine (Goese et al., 1999). These are formed *via* transamination of BCAAs, followed by oxidative decarboxylation of the ketoacid intermediate (scenario 1) (Goese et al., 1999; Xu et al., 2013; Mishra et al., 2020). BCAA catabolism followed by elongation of α-keto acids could produce a range of short to medium chain acyl-CoAs, including the PC precursors hexanoyl-CoA and butanoyl-CoA (**Supplementary Figure S1**). Another possible route may involve *de novo* FA biosynthesis (scenario 2). This pathway could impact the pool of available acyl-CoAs either directly by contributing to the synthesis of short to medium acyl-CoAs, or indirectly *via* the synthesis of longer carbon chain FAs which are then broken down to form PC precursors (scenario 3) (Marks et al., 2009). Notably, a β-ketoacyl reductase (KAR; 3-oxoacyl-[acyl-carrier-protein] reductase; EC 1.1.1.100) is closely associated with PC biosynthesis based on association mapping in a PC alkyl side chain diversity panel and its co-expression with a cytosolic hexanoyl-CoA synthetase (e.g., AAE) gene (Stout et al., 2012; Zager et al., 2019; Welling et al., 2020). For scenario 3, an oxylipin biosynthesis origin is predicted, whereby FAs are degraded into smaller carbon chains following oxidation and lyase reactions (**Supplementary Figure S1**), with genes encoding a linoleate 13*S*-lipoxygenase (LOX) and hydroperoxide lyase (HPL) highly expressed in glandular trichomes of *C. sativa* (Marks et al., 2009; Stout et al., 2012; Livingston et al., 2020); the focus of the present study.

Phyto-oxylipins are a large family of oxidized lipid-derivatives that can act as signaling molecules and play a pivotal role in regulating developmental processes, such as wound healing and plant defense. Jasmonic acid (JA) is one of the most studied oxylipins in plants (Blée, 2002; Ghorbel et al., 2021). Oxylipin biosynthesis starts with the oxidization of free FAs, such as either linoleic acid (C_18:2_) or linolenic (C_18:3_) acid, forming a hydroperoxy FA. This is catalyzed by either 9*S*- or 13*S*-LOX which incorporate molecular oxygen at C_9_ or C_13_, respectively (Feussner and Wasternack, 2002). Hydroperoxy products are then metabolized by members of the Cyp74-family (cytochrome P450 family), including allene oxide synthase (AOS) which forms the JA precursor 12,13(*S*)-epoxy-octadecatrienoic acid (12,13-EOT) (Song and Brash, 1991). HPL is another Cyp74-family member, and this sub-branch of oxylipin biosynthesis forms ω-oxo FAs, including the wounding hormone traumatin, as well as green leafy volatiles (GLV) such as C_6_ aldehydes by cleavage of the C-C bonds in the hydroperoxide of LOX products (Howe et al., 2000). It is hypothesized that C_6_ volatiles resulting from 13*S*-LOX and HPL activity could act as progenitors to hexanoyl-CoA in PC biosynthesis (Stout et al., 2012; Livingston et al., 2020).

Upregulation of the lipoxygenase pathway following exogenous exposure to the oxylipin wound hormone and volatile JA methyl ester methyl jasmonate (MeJA) has been widely reported in various plant organs and tissues, including in developing barley (*Hordeum vulgare*) grains, tomato (*Solanum lycopersicum*) leaves, maize (*Zea mays*) seedlings and lupin (*Lupinus luteus*) inflorescences (Heitz et al., 1997; Rouster et al., 1997; Kim et al., 2003; Kućko et al., 2022). To test the hypothesis that LOX activity and oxylipin-mediated aldehyde production could contribute to polyketide FA starter units, we examined the impact of MeJA on a *C. sativa* chemotype capable of producing both C_5_ and C_3_ alkyl side chain PC analogues. We used targeted profiling of 14 PCs and qPCR analysis of the putative candidate genes *LOX-L* and *HPL*, as well as an untargeted metabolomic analysis by ultra-high-performance liquid chromatography–heated electrospray ionization high-resolution mass spectrometry (UHPLC-HESI/HRMS) to improve our understanding of the relationship between oxylipin and PC biosynthesis.

## 2 Materials and methods

### 2.1 Plant materials

All research activities, including the procurement and cultivation of *C. sativa* (industrial hemp) germplasm, were performed in accordance with Part IVA of the Drugs, Poisons and Controlled Substances Act 1981, and under an authorization issued by Agriculture Victoria, Department of Jobs, Precincts and Regions (DJPR), Victorian State Government, Australia.

A single female genotype (MW6-15) derived from an industrial hemp line (Accession #6) was used for analysis (Welling et al., 2021). Fifteen cuttings were taken from this genotype and rooted in Grodan^®^ rockwool propagation cubes (36 mm x 36 mm x 40 mm) for a period of 21 d. Rooted cuttings were transplanted into 400 mL pots with soil media comprising of a 1:1:1 (w/w/w) perlite: peat moss: vermiculite mix as well as dolomite (1.1 g/L). Cuttings were grown vegetatively for 14 d in a controlled environment room (CER) at 24 °C (55% humidity) under 18-h light/6-h darkness. Plant clones were then transplanted into 8 L pots containing the aforementioned soil media and transferred to short-d conditions (12-h light/12-h darkness) to initiate floral development. During long-d photoperiod growth (vegetative) plants were watered daily using RO water supplemented with CANNA^®^ Classic Vega (4 mL/L CANNA Vega A and 4 mL/L CANNA Vega B in RO water) nutrient solution and then with CANNA^®^ Classic Flores (4 mL/L CANNA Flores A and 4 mL/L CANNA Flores B in RO water) nutrient solution under short-d conditions (flowering) (**Supplementary Table S1**). Light was provided by low-pressure mercury discharge lamps (Philips TL-D 58 W, Amsterdam, Netherland), delivering a light intensity (PPFD) of 360 (μmol m-^2^ s-^1^) at 35 cm from the light source.

### 2.2 Reagents and standards

Reagent and analytical standard preparation followed (Welling et al., 2021). For identification purposes, olivetolic acid (OA) was purchased from Novachem Pty Ltd (Heidelberg West, Vic., Australia), while JA, 12-oxo-Phytodienoic acid (OPDA), and Jasmone were purchased from Sapphire Bioscience Pty Ltd (Redfern, NSW, Australia). MeJA was purchased from Sigma-Aldrich (St. Louis, MO, USA). Δ9-THC-d3 (2 µg/mL) purchased from Novachem Pty Ltd (Heidelberg West, Vic., Australia) was used as an internal standard.

### 2.3 Methyl jasmonate (MeJA) and neomycin treatments

Fourteen d after transfer to a short-d photoperiod and on the onset of terminal flowering, defined here as meristem termination with an apical female flower, plant clones were subject to either one of five treatments: 0 mM MeJA (control), 1 mM MeJA, 7.5 mM MeJA, 15 mM MeJA, or 0.5 mM neomycin (negative control) dissolved in RO water supplemented with 0.8% (v/v) EtOH and 0.1% (v/v) Tween 20. Solutions were applied as a whole-plant foliar spray. Three biological replicates (plant clones) were used per treatment, and each biological replicate received 25 mL of the spray solution. Treatments were applied in a separate growing area to prevent cross contamination, and these were administered on three occasions over a 14-d period, with plant material harvested 24 h after the final treatment or 29-d post-exposure to a short-d photoperiod. Plant material taken from the apical (top 30 cm) inflorescence was snap-frozen in liquid nitrogen and stored at -80 °C. Foliage leaves of the 1^st^ - 3^rd^ order were manually removed prior to collection to enrich for trichome-containing vegetative tissues and reproductive organs: bracts and flowers, respectively. The complexity and compactness of *C. sativa* inflorescence architecture complicated the development of a reproducible wounding treatment and was therefore omitted from our experiment.

### 2.4 Sample preparation for mass spectrometry analysis

Inflorescence material enriched for bracts and flowers (~6 g) was freeze-dried for 7 d. After drying, the material was manually passed through a fine-mesh (250 μm) sieve to remove stems. Floral material was homogenized using a Geno/Grinder 2010 at 1500 rpm for 2 × 30 s intervals within 15 mL polycarbonate vials containing 2 x stainless steel grinding balls. The finely ground floral tissue (50 mg) was weighed into 5 mL Eppendorf Safe-Lock microcentrifuge tubes and extracted in 5 mL LCMS-grade MeOH containing Δ9-THC-d3 (2 µg/mL; internal standard) by vortex mixing for 1 min, followed by sonication for 20 min at room temperature. Particulate material was allowed to sit for a minimum of 3 min and then filtered using a 0.45 μm polytetrafluoroethylene (PTFE) syringe filter. Sample extracts were diluted 40 x with MeOH containing Δ9-THC-d3 (2 µg/mL) and aliquots of the neat and diluted filtrate were transferred to amber HPLC vials (2 mL) and stored at –80°C prior to analysis.

### 2.5 Liquid chromatography-high-resolution mass spectrometry analysis

UHPLC-HESI/HRMS analysis was as described (Welling et al., 2021), with minor modifications. Briefly, sample runs were carried out on a Thermo Fisher Vanquish Flex UHPLC system with solvent degasser, quaternary pump, temperature-controlled sampler/auto injector and column compartment, and photodiode array detector (DAD) coupled to an Orbitrap ID-X Tribrid high resolution mass spectrometer (Thermo Fisher Scientific Inc., MA, USA). Chromatographic separation was performed using a Phenomenex Kinetex C18 column, 1.7 μm, 150 mm × 2.1 mm (Phenomenex Australia Pty Ltd, NSW, Australia). The mobile phase A was water with 0.1 % (v/v) formic acid and the mobile phase B was acetonitrile with 0.1 % (v/v) formic acid. The following gradient program was used: 10 % B, 0–2 min; 10 to 40 % B, 2–3 min; 40 % B, 3–5 min; 40 to 80 % B, 5–6 min; 80 % B, 6–9 min; 80 to 90 % B, 9–11 min; 90 to 100 % B, 11–12 min; 100 % B, 12–15 min; 100 to 10 % B, 15–16 min; and 10 % B from 16–20 min.

MS was operated using a HESI interface in positive and negative ion modes. For MS and MS^2^, the orbitrap was the mass analyzer used. Pierce FlexMix calibration solution was used to calibrate the MS prior to data acquisition, and the internal mass calibrant fluoranthene (Easy-IC) was activated for real-time mass calibration during data acquisition. Xcalibur (v4.4) software (Thermo Fisher Scientific Inc., MA, USA) was used for data acquisition. For quantitative analysis, Trace Finder (v4.1) software (Thermo Fisher Scientific Inc., MA, USA) was used on full MS data. Integration of extracted ion peaks and comparison of retention time with reference standards was used for peak identity. Δ9-THC-d3 (2 µg/mL) was used as an internal standard, and concentration of each analyte was determined by interpolation from standard calibration curves generated in MS Excel. Results were expressed as average concentration ± standard deviation (s.d.) from duplicate extraction replicates.

### 2.6 Compound annotation

Small-molecule identification was carried out using Thermo Scientific Compound Discoverer (v3.3) software. The following parameters were used for compound detection and alignment of retention times: mass tolerance [ppm] = 5; min. Peak Intensity: 50 000; Chromatographic S/N Threshold: 1.5; Ions (positive mode): [2M+H]^+1^, [2M+K]^+1^, 2M+Na]^+1^, [M+H]^+1^, [M+H-H2O]^+1^, M+K]^+1^, [M+Na]^+1^; Base Ions (positive mode): [M+H]^+1^; [M+H-H2O]^+1^. Ions (negative mode): [2M-H]^-1^, [M-2H]^-2^, M-2H+K]^-1^, M-H]^-1^, [M-H-H2O]^-1^; Base Ions (negative mode): [M-H]^-1^; alignment model: adaptive curve; maximum shift [min]: 2; mass tolerance: 5 ppm; min. element counts: C H; max. element counts = C_100_ H_300_ Br_5_ Cl_10_ N_20_ Na_5_ O_50_ P_10_ S_10_. To correct for batch effects and reduce drift, peak areas were quality control (QC)-corrected using a QC sample developed by pooling inflorescence samples across all treatments. These were measured after every 15 injections.

Annotation of putative compounds was conducted using calculated elemental composition and searches with high-resolution accurate mass (HRAM) MS^n^ spectral library mzCloud, as well as compound databases (ChemSpider and Metabolika) and ranking (mzLogic) algorithmic tools, along with fragment ion searching (FISh) to predict *in silico* fragmentation. The following parameters were used for mzCloud searches: compound classes = all; precursor mass tolerance: 10 ppm; FT fragment mass tolerance: 10 ppm; IT fragment mass tolerance [Da] = 0.4; library = auto processed, reference; post processing = recalibrated; max # results = 10; annotate matching fragments = true. Search parameters for DDA were as follows: identity search: HighChem HighRes; match activation type: true; match activation energy: match with tolerance; activation energy tolerance: 20; apply intensity threshold: true; similarity search: confidence forward; match factor threshold: 60. The following databases were included in ChemSpider searches: Cambridge Structural Database, Carotenoids Database, ChEBI, ChEMBL, DrugBank, KEGG, LipidMAPS, NIST Chemistry WebBook, Phenol-Explorer, PlantCyc, PubMed. Database searches were performed by mass and formula using the following parameters: mass tolerance: 5 ppm, max. # of results per compound: 100; max. # of predicted compositions to be searched per compound: 3; The following parameters were used for mzLogic: FT fragment mass tolerance: 5 ppm; IT fragment mass tolerance: 0.4 Da; max. # compounds: 0; max. # mzCloud similarity results to consider per compound: 10; match factor threshold: 30.

The annotation process corresponded to level 2 of confidence as set out by the COordination of Standards in MetabOlomicS (COSMOS) and the Chemical Analysis Working Group (CAWG) Metabolomics Standards Initiative (MSI) (Sumner et al., 2007). The compound list was restricted to compounds with chromatographic peak areas > 50 000 as well as those with MS^2^ fragmentation spectra using the following filtering parameters: background is false; annot.source has status full match in source predicted compositions; group area is greater than 50 000 in any sample group; norm. area has any value in any file; MS^2^ is equal to DDA for preferred ion. Putatively annotated compounds from this list were used for co-expression network construction and metabolomic comparisons between control and treated plants.

### 2.7 Gene expression analyses

Snap frozen inflorescence material (~2 g) enriched for bracts and flowers was ground using a Geno/Grinder 2010 at 1500 rpm for 2 × 30 s intervals within 15 mL polycarbonate vials containing 2 x stainless steel grinding balls. The Spectrum Plant Total RNA kit (Sigma-Aldrich) was used to isolate total RNA from homogenized inflorescence material (~100 mg fresh weight), with DNase I on-column digestion performed in accordance with the manufacturer’s instructions. DNA-free RNA was eluted in 30 µL of RNase-free water. RNA concentrations were determined using a Qubit 4 Fluorometer and Qubit RNA BR Assay Kit (Thermo Fisher Scientific, USA), with RNA integrity assessed by gel electrophoresis. RNA was extracted from three biological replicates (plant clones) per treatment (0 mM MeJA (control), 1 mM MeJA, 7.5 mM MeJA, 15 mM MeJA, or 0.5 mM neomycin). Single strand cDNA was synthesized using SuperScript IV VILO Master Mix with ezDNase Enzyme (Thermo Fisher Scientific, USA) and diluted 1:5 to a total RNA equivariant of 10 ng/µL.

Quantitative PCR with threshold cycle (Cq) determination was performed using a fluorescence baseline setting of 0.3 (QuantStudio 5 Real-Time PCR System, Applied Biosystems). A total of 2.5 µL of 8 × diluted cDNA was used for quantitative PCR in a total volume of 10 µL with SYBR Select Master Mix (Applied Biosystems). Data were normalized by using a previously validated reference gene *EF1α* (Riedel et al., 2014; Guo et al., 2018). PCR efficiencies as well as the sensitivity and robustness of the assay were determined by means of calibration curves, with PCR efficiencies calculated using the formula: PCR efficiency = 10^−1/slope^ – 1 (Bustin et al., 2009). Calibration curves were generated for each target gene primer pair (**Supplementary Table S2**). PCR products were amplified using Phusion High-Fidelity PCR Master Mix with HF Buffer (Thermo Fisher Scientific, USA) and amplicons sequence-verified at the Australian Genome Research Facility (AGRF). Data were expressed as 40-ΔCq, where ΔCq is the difference between the Cq of the target gene and reference gene. As such, a 40-ΔCq value equal to 40 represents a transcript amount equal to the transcript abundance for the reference gene.

### 2.8 Statistical analysis

One-way analysis of variance (ANOVA) with *post-hoc* Dunnett’s multiple comparisons test of control (0 mM) vs ≥ 1 MeJA and neomycin treatments and one-way ANOVA test for trend were performed using GraphPad PRISM (v9.1.0) software (GraphPad Software, Inc. San Diego, CA, USA). Analyses were performed on three biological replicates (plant clones) per treatment group. For untargeted metabolomic analyses, QC-corrected chromatographic peak areas were mean-centered and log2-transformed. Principle component and hierarchical cluster analysis (HCA) using Euclidean distance were conducted using MetaboAnalyst (v5.0) software. Signed, weighted correlation networks were produced in R (v3.6.3), with the R package WGCNA (v1.70.3) (Langfelder and Horvath, 2008), with a soft thresholding power of 20 (positive mode) and 12 (negative mode). Network visualization was performed using VisANT (Hu et al., 2008). Kyoto Encyclopedia of Genes and Genomes (KEGG, website: https://www.genome.jp/kegg/pathway.html) pathway overrepresentation (enrichment) analysis was performed by the Metabolites Biological Role (MBRole) server (López-Ibáñez et al., 2016) using KEGG compound IDs retrieved from Compound Discoverer software (v3.3). The background set was set to ‘full database’ and was based on all the compounds in the database associated with the selected annotations. Enrichment *p*-values were adjusted for multiple testing using the Benjamini–Hochberg correction for the false discovery rate (FDR) (Benjamini and Hochberg, 1995).

## 3 Results

### 3.1 *C. sativa plants* are morphologically impacted by MeJA treatment

**Figure 1:**
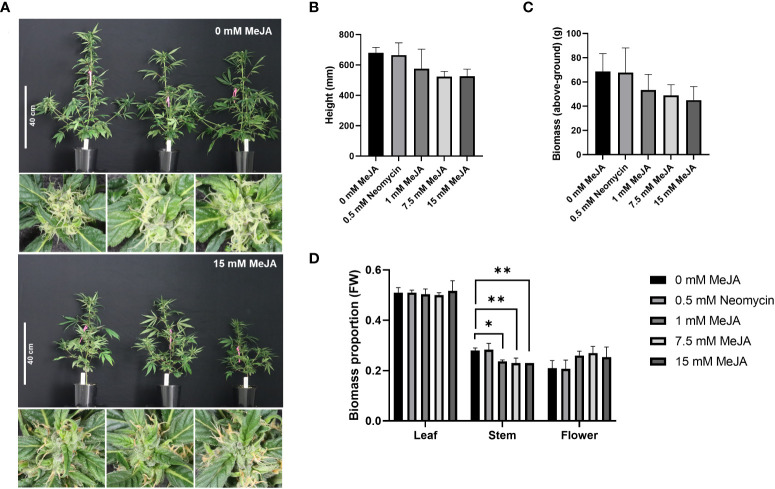
Impact of MeJA and neomycin treatment on *Cannabis sativa* plant morphology. **(A)** Visible reductions in plant height and amber coloration of stigmas observed with MeJA application. **(B)** Impact of MeJA on plant height. Mean (*n* = 3) ± SD. **(C)** Impact of MeJA on above-ground biomass. Mean (*n* = 3) ± SD. **(D)** Proportion of leaf, stem and flower from the total above-ground biomass of MeJA and neomycin treated plants. Mean (n=3) ± SD. One-way ANOVA Dunnett’s multiple comparisons test of control (0 mM) vs neomycin and ≥ 1 MeJA treatments *p< 0.05, **p < 0.01; One-way ANOVA test for trend is significant for height and biomass (*p* < 0.05); Three biological replicates (plant clones) used per treatment; Plant measurements taken 29-d post exposure to a short-d photoperiod; Close-up images of pistillate flowers taken from the apical inflorescence.

To investigate the impact of methyl jasmonate (MeJA) on phytocannabinoid (PC) biosynthesis, flowering plant clones from an industrial hemp genotype were subjected to whole-plant foliar applications of varying MeJA concentrations and harvested and analyzed as described in the Material & Methods section. The morphology of plant clones was visibly impacted following exogenous MeJA exposure, with differences in plant stature as well as stigma coloration observed at harvest (**Figure 1A**). A reduction in plant height at harvest was most evident in the 7.5- and 15-mM MeJA treatments, with > 80% of stigmas of these plants exhibiting an amber hue 24 h post treatment (**Figures 1A, B**). No obvious differences in plant stature were found among the control (0 mM MeJA) and neomycin-treated plants and stigma coloration was unaffected in either of these treatments (**Figures 1A, B**). A reduction in above-ground biomass was also apparent among the MeJA treatments compared to the control plants (**Figure 1C**). While no statistically significant differences in plant height and above-ground plant biomass were found among the treatments (one-way ANOVA, Dunnett’s test, *p* = > 0.05) (**Figures 1B, C**), the one-way ANOVA *p*-value of 0.07 for height was close to being statistically significant. There was also a significant linear trend of both decreasing height and above-ground biomass with increasing MeJA molar concentrations (one-way ANOVA trend test, *p* < 0.05). A difference in partitioning of above-ground biomass into flower and stem was also apparent among the treatments (**Supplementary Table S3**). Significant decreases in the proportion of stem biomass in the 1-, 7.5- and 15-mM MeJA treatments (*p* < 0.05) were observed, while the proportion of flower biomass tended to increase with increasing MeJA concentration (**Figure 1D**; **Supplementary Table S3**). No obvious changes in trichome density or morphology were found among the control and treated plants in the foliage leaves, reduced floral leaves and perigonal bracts when assessed by light microscopy (**Supplementary Figure S2**).

### 3.2 MeJA treatments increase PC content in a dose-dependent manner

**Figure 2:**
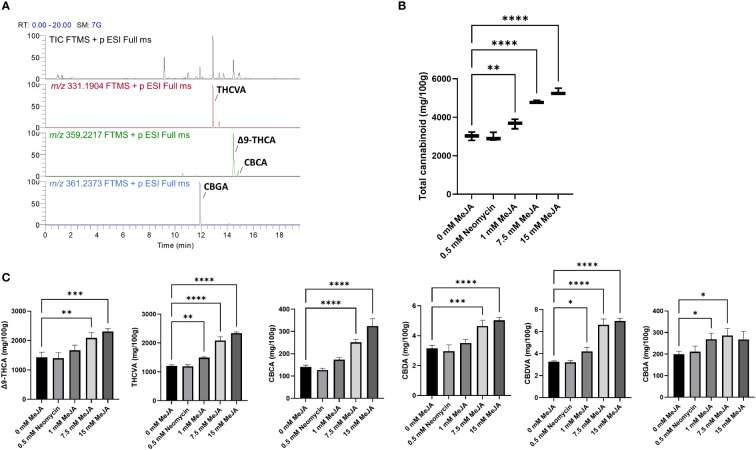
PC content of MeJA and neomycin treated plants. **(A)** Total ion chromatogram (TIC) and extracted ions *m/z* 331.1904 and *m/z* 359.2217 of a representative methanol extract of the floral tissues of Accession # 6 (genotype MW6-15). CBGA, THCVA, Δ9-THCA and CBCA are the most abundant phytocannabinoids. **(B)** The response of total acidic PC content to MeJA and neomycin treatment. **(C)** The changes in the concentrations of individual PC contents in response to MeJA and neomycin treatment. Mass tolerance of extracted ions ≤ 5 ppm. One-way ANOVA Dunnett’s multiple comparisons test of control (0 mM) vs neomycin and ≥ 1 MeJA treatments *p < 0.05, **p < 0.01, ***p < 0.001, ****p < 0.0001.

To determine the impact of MeJA treatment on PC content and composition, 14-d-old inflorescences with 1^st^ - 3^rd^ order foliage leaves removed were subject to targeted quantitative PC profiling. The industrial hemp genotype used for this analysis exhibited a chemotype high in the C_5_ alkyl side chain PC Δ9-tetrahydrocannabinolic acid A (Δ9-THCA) and C_3_ alkyl side chain PC Δ9-tetrahydrocannabivarinic acid (THCVA), as can be seen from the total ion chromatogram and extracted ion chromatograms *m/z* 331.1904 and *m/z* 359.2217 (**Figure 2A**). PCs were predominantly in their acidic forms, with neutral PC dry weight concentrations (w/w) orders of magnitude lower than their carboxylic acid precursors (**Supplementary Table S4**). Other acidic PC species could also be quantified using the extracted ion chromatogram data in conjunction with known retention times of standards, including cannabichromenic acid (CBCA) and cannabigerolic acid (CBGA) as well as cannabidiolic acid (CBDA) and cannabidivarinic acid (CBDVA), although these were at much lower concentrations than Δ9-THCA or THCVA (**Supplementary Table S4**).

To understand the overall effect of MeJA on PC content, PC values were combined to give a total dry weight (w/w) in the inflorescences of MeJA and neomycin-treated plants. Significant increases in PC content were found in the 1 mM MeJA (*p* < 0.01) as well as in the 7.5- and 15-mM MeJA (*p* < 0.001) treatments when compared with the 0 mM MeJA control (**Figure 2B**). PC content was found to increase in a dose-dependent manner, with an almost two-fold increase in PC content measured in the 15 mM MeJA treated plants. The dry weight (w/w) content of individual PCs followed a similar pattern for both major and minor acidic PCs, with significant increases in PC content in the 7.5- and 15-mM MeJA treatments (**Figure 2C**). The exception was CBGA, whose content was significantly higher in both the 1- and 7.5-mM treatments, but not the 15 mM treatment (**Figure 2C**). No significant differences in PC content were observed for the neomycin-treated group (**Figures 2B, C**).

### 3.3 Composition of alkyl side chain analogues is impacted by MeJA

**Figure 3:**
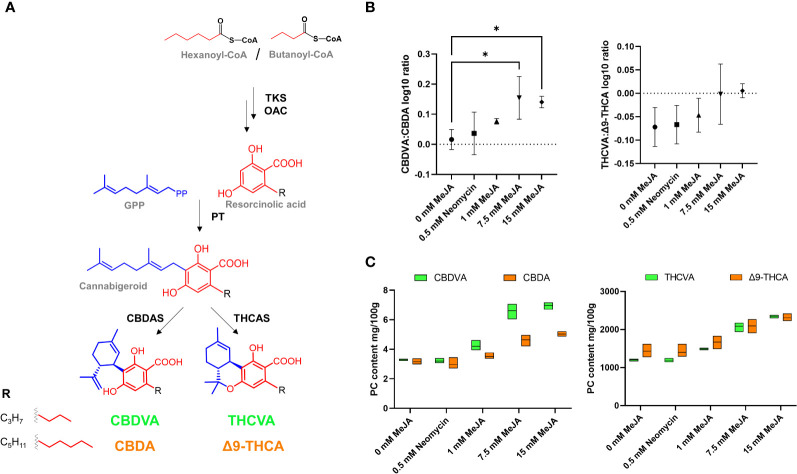
Composition of PC alkyl side chain analogues following MeJA and neomycin treatment. **(A)** PC biosynthesis pathway. **(B)** Comparison of C_3_:C_5_ PC alkyl side chain analogue log10 ratio. **(C)** PC content of alkyl side chain analogues CBDVA (C_3_), CBDA (C_5_), THCVA (C_3_) and Δ9-THCA (C_5_). One-way ANOVA Dunnett’s multiple comparisons test of control (0 mM) vs neomycin and ≥ 1 MeJA treatments *p < 0.05. One-way ANOVA test for trend is significant for CBDVA : CBDA ratio and THCVA: Δ9-THCA ratio (*p* < 0.05). CBDA - cannabidiolic acid; CBDAS - cannabidiolic acid synthase (EC:1.21.3.8); CBDVA - cannabidivarinic acid; OAC - olivetolic acid cyclase (EC:4.4.1.26); PT - geranylpyrophosphate:olivetolate geranyltransferase (EC 2.5.1.102); THCAS - Δ9 tetrahydrocannabinolic acid synthase (EC:1.21.3.7); THCVA - Δ9-tetrahydrocannabivarinic acid; TKS - tetraketide synthase (EC:2.3.1.206); Δ9-THCA - Δ9 tetrahydrocannabinolic acid.

The use of a *C. sativa* chemotype capable of producing high levels of both C_3_ and C_5_ alkyl side chain PCs allowed for the examination of the impact of MeJA on alkyl side chain analogue composition. The length of the alkyl side chain of PCs is directed by the availability of acyl-CoA starter units (Luo et al., 2019). The use of hexanoyl-CoA during polyketide assembly results in the formation of C_5_ alkyl side chain analogues CBDA and Δ9-THCA, while assembly with butanoyl-CoA would result in the formation of C_3_ alkyl side chain analogues CBDVA and THCVA (**Figure 3A**). Significant increases in the CBDVA : CBDA ratio were observed at the 7.5- and 15-mM MeJA treatments (one-way ANOVA, Dunnett’s test, *p* < 0.05), and a significant linear trend of increasing C_3_:C_5_ alkyl ratios with increasing MeJA molar concentrations was also found among the Δ9-THC(V)A-type subclasses (one-way ANOVA, trend test, *p* < 0.05) (**Figure 3B**). For the control and neomycin treatments, CBDVA and CBDA levels were close to parity, with increases in CBDVA exceeding those of CBDA at the 1-, 7.5- and 15-mM MeJA treatments (**Figure 3C**). Levels of THCVA also increased at a faster rate than for Δ9-THCA, with THCVA and Δ9-THCA becoming close to parity at the 7.5- and 15-mM MeJA treatments (**Figure 3C**).

### 3.4 Metabolomic profiles differ among MeJA treated plants

To further investigate the impact of MeJA treatment on PC biosynthesis, we compared the metabolite profile of inflorescence samples across treatments using an untargeted metabolomic approach by UHPLC-HESI/HRMS with data-dependent MS^2^ acquisition in both positive and negative ion modes. In the positive ion mode, analysis across all treatments identified 137,196 features that accounted for 6,398 putative compounds distinguishable by retention time and molecular weight. In the negative ion mode 78,852 features were detected, and these accounted for 3,778 putative compounds. After filtering by eliminating low abundant chromatographic peak areas < 50 000 counts and those without MS^2^ fragmentation spectra, 1,224 (positive ion mode) and 769 (negative ion mode) compounds remained in the list (**Supplementary Table S5**).

**Figure 4:**
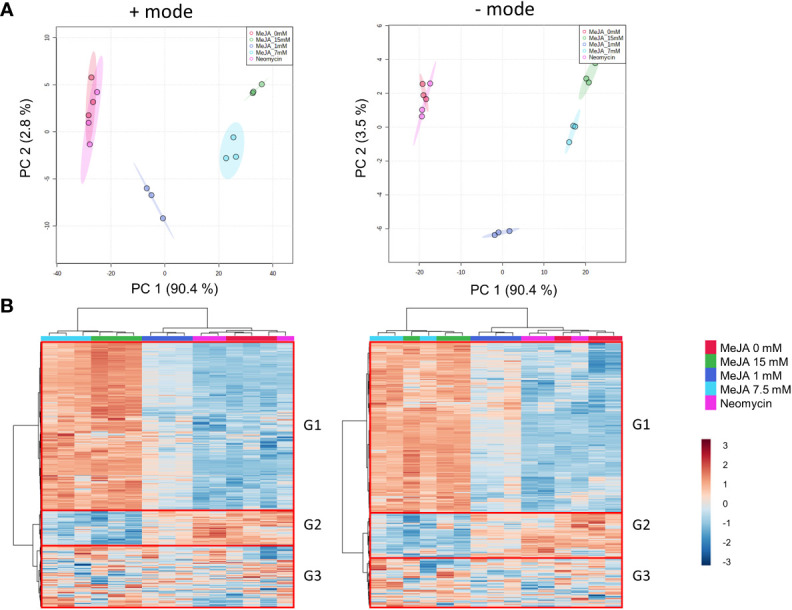
Metabolome effects of MeJA and neomycin treatment on inflorescence. **(A)** PCA of quality control-corrected metabolite chromatographic peak areas in positive and negative modes. **(B)** HCA and heatmap of quality control-corrected metabolite chromatographic peak areas in positive and negative modes. G1-G3 represent the major metabolite groups by abundance profile across samples. *Red boxes* indicate the three main metabolite groups. Median values from duplicate extraction replicates used per biological replicate. Hierarchical clustering, heatmap and PCA performed on 1,224 (positive mode) and 769 (negative mode) putative metabolites. For multivariate analysis, mean-centred normalization and log2 transformation scaling were applied to quality control-corrected metabolite abundances.

Results from the multivariate analysis of the methanol-soluble metabolomes of MeJA and neomycin treatments were comparable in both positive and negative ion modes (**Figures 4A, B**). Principal component analysis (PCA) showed a clear separation between the 1-, 7.5- and 15-mM MeJA treatments, with the first two principal components accounting for ~ 90% (first principal component) and ~ 3% (second principal component) of the variability in the dataset, respectively (**Figure 4A**). Biological replicates of the 0 mM MeJA control and neomycin treatments clustered together, indicating that the neomycin treatment had negligible impact on the metabolite profile of these plants. Hierarchical clustering analysis (HCA) of inflorescence samples formed two major clades (**Figure 4B**), one comprising the 0 mM MeJA control, 1 mM MeJA and neomycin treatment samples and the other the 7.5- and 15-mM MeJA treatments (**Figure 4B**). This suggests that the inflorescences treated with the high molar MeJA concentrations have distinct methanol-soluble metabolomes compared to the neomycin, 0 mM and 1 mM MeJA samples.

Three main groups can be distinguished from the heatmap of putative compounds. Group 1 (top, G1), which accounts for approximately two-thirds of all metabolites evaluated, has high chromatographic peak area values in the 7.5- and 15-mM MeJA treatments, followed by intermediate values in the 1 mM MeJA treatment and low peak area values in the 0 mM MeJA control and neomycin treatments (**Figure 4B**). Group 2 (middle, G2) which is a smaller group and has the opposite chromatographic peak area values (high values in the 0 mM MeJA control and neomycin treatments and low values in the 7.5-and 15-mM MeJA treatments), and Group 3 (bottom, G3) which has peak area values unrelated to the treatment groups (**Figure 4B**).

### 3.5 Metabolite co-expression modules correspond to PC content

To gain an understanding of the cellular responses to exogenous MeJA treatments and its impact on PC biosynthesis, weighted gene co-expression network analysis (WGCNA) was used. Originally developed for high-throughput microarray and RNA-seq analyses, WGCNA can also be applied to metabolomic datasets for meaningful biological interpretation (Pei et al., 2017). Using chromatographic peak area abundances from MS data in positive and negative ion modes, WGCNA was employed to identify correlative relationships between metabolites across MeJA-treated samples by clustering highly correlated metabolites into modules and examining their correlations to chemotypes scored through targeted PC profiling.

**Figure 5:**
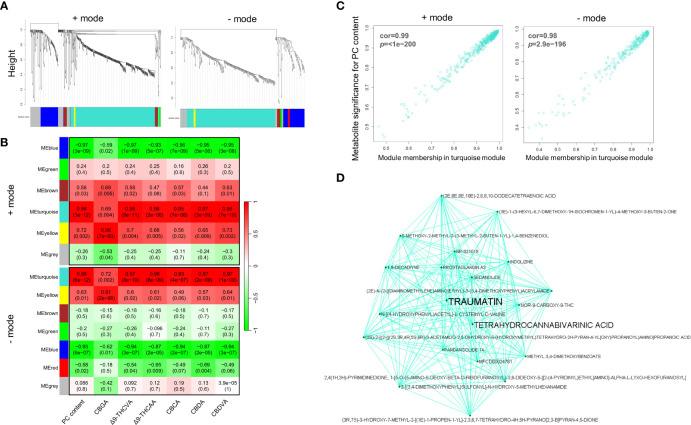
Weighted correlation network analysis of inflorescence metabolomes. **(A)** Clustering dendrograms of putative metabolites from UHPLC-HESI/HRMS analysis with assigned module colours. Dissimilarity based on topological overlap. **(B)** Module–chemotype associations. Rows correspond to a module eigenmetabolites, and columns correspond to the dry weight (w/w) PC content. The correlation and *p*-value are displayed in each cell in parenthesis. **(C)** Scatter plot of metabolite significance for PC content vs turquoise module membership and **(D)** visualization of the network connections among the most connected genes in the turquoise module. Module membership (MM) represents the intramodular connectivity of a metabolite in each module. A high MM indicates that a metabolite has a high correlation with the module eigenmetabolites.

Weighted co-expression network construction identified 13 modules (six from positive mode, seven from negative mode), as can be visualized from the hierarchical clustering dendrograms together with assigned module colors in **Figure 5A**. To identify modules that are significantly associated with PC biosynthesis, module eigenmetabolites (ME) were correlated with PC content (**Figure 5B**). Module–chemotype correlations identified that the eigenmetabolites of the turquoise modules from positive and negative ion modes showed strong positive correlations with PC content (*r* = 0.99), while eigenmetabolites of the blue modules from positive (*r* = -0.97) and negative (*r* = -0.93) ion modes showed strong negative correlations with PC content (**Figure 5B**; **Supplementary Tables S6**, **S7**). These modules corresponded to Group 1 (turquoise modules) and Group 2 (blue modules) from the HCA analysis (**Figures 4B**, **5A**), with the eigenmetabolites of the turquoise modules having the highest abundance at 7.5-and 15-mM MeJA treated samples while the eigenmetabolites of the blue modules having the lowest abundances at these concentrations. There were significant differences in the enrichment of KEGG pathways between the turquoise and blue modules (**Table 1**). In the blue negatively correlated modules, “Alanine, aspartate and glutamate metabolism (map00250)”, “D-Glutamine and D-glutamate metabolism (map00471)” and “Arginine and proline metabolism (map00330)” were the most significantly enriched pathways, while in the turquoise positively correlated modules the KEGG pathways “Biosynthesis of plant hormones (map01070)”, “Phenylalanine, tyrosine and tryptophan biosynthesis (map00400)” and “Biosynthesis of secondary metabolites (map01110)” were the most significantly enriched (**Table 1**).

**Table 1:** KEGG pathway enrichment analysis of modules correlated to PC content.

Ion mode	Modules	KEGG pathway	P-value	FDR correction
–	Turquoise	Biosynthesis of plant hormones (map01070)	1.69E-04	3.09E-03
+	Turquoise	Phenylalanine, tyrosine and tryptophan biosynthesis (map00400)	1.19E-04	4.66E-03
+	Turquoise	Biosynthesis of secondary metabolites (map01110)	2.55E-04	4.96E-03
–	Turquoise	Metabolic pathways (map01100)	1.93E-03	1.35E-02
+	Turquoise	Aminoacyl-tRNA biosynthesis (map00970)	2.47E-03	1.47E-02
+	Turquoise	Biosynthesis of alkaloids derived from shikimate pathway (map01063)	1.41E-03	1.47E-02
+	Turquoise	Biosynthesis of plant hormones (map01070)	1.86E-03	1.47E-02
+	Turquoise	Glucosinolate biosynthesis (map00966)	2.37E-03	1.47E-02
+	Turquoise	Biosynthesis of phenylpropanoids (map01061)	5.12E-03	2.50E-02
–	Blue	Alanine, aspartate and glutamate metabolism (map00250)	4.10E-07	2.05E-05
–	Blue	D-Glutamine and D-glutamate metabolism (map00471)	3.93E-06	9.83E-05
+	Blue	Alanine, aspartate and glutamate metabolism (map00250)	2.74E-05	6.27E-04
+	Blue	Arginine and proline metabolism (map00330)	4.32E-05	6.27E-04
–	Blue	Nitrogen metabolism (map00910)	4.54E-05	7.57E-04
–	Blue	Biosynthesis of phenylpropanoids (map01061)	1.19E-04	1.34E-03
–	Blue	Metabolic pathways (map01100)	1.34E-04	1.34E-03
+	Blue	Metabolic pathways (map01100)	3.60E-04	3.48E-03
–	Blue	Aminoacyl-tRNA biosynthesis (map00970)	1.09E-03	6.80E-03
–	Blue	Flavonoid biosynthesis (map00941)	8.17E-04	6.80E-03
–	Blue	Biosynthesis of secondary metabolites (map01110)	2.99E-03	1.50E-02

### 3.6 Identification of hub metabolites

Consistent with the enrichment of secondary metabolite and plant hormone biosynthesis pathways, metabolites involved in oxylipin, and PC biosynthetic pathways are overrepresented in the turquoise module. Among the metabolites within the positively correlated turquoise modules were the PCs Δ9-THCA, THCVA, CBCA, CBDA, CBDVA, and CBGA, as well as the PC resorcinyl intermediate OA, and these compounds matched the nominal mass (Δ ppm < 5) and retention times of certified PC reference standards (highlighted in black bold in **Supplementary Tables S6**, **S7**). Other putative PCs were also identified among these modules, but these were not verified using reference standards. As evidence of the robustness of the module assignments, CBGA was among the metabolites present in the yellow module positively correlated with CBGA content (*r* = 0.96, *p*-value 0.002) (**Figure 5B**). Several jasmonate (JA)-related compounds were also annotated within the turquoise modules by consensus evaluation based on elemental composition prediction, spectral library (mzCloud) and database (ChemSpider) searches. These include JA, MeJA, 12-oxophytodienoic acid (OPDA), and the JA derivative jasmone (highlighted in blue bold in **Supplementary Tables S6**, **S7**), and these compounds were confirmed by comparison with certified reference standards. In addition, metabolites that have a high significance for PC content also showed a high module membership (MM) in the turquoise modules for both the positive and negative ion modes (**Figure 5C**). Taken together, these data suggest that the eigenmetabolite of the turquoise modules demonstrates a strong relationship between the oxylipin and PC biosynthesis pathways.

**Figure 6:**
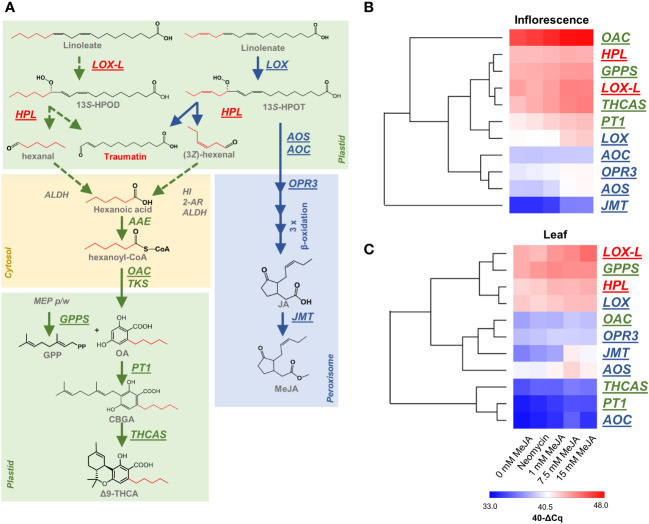
Responsiveness of PC and oxylipin biosynthesis genes to MeJA and neomycin treatment. **(A)** Schematic representation of PC and oxylipin biosynthetic pathways and their potential interactions. **(B)** The relative abundance of PC and oxylipin biosynthesis genes in the inflorescences of MeJA and neomycin treated plants. **(C)** The relative abundance of PC and oxylipin biosynthesis genes in the leaves of MeJA and neomycin treated plants. Underlined genes (shown in **A**) have been assessed by qPCR analysis with *EF1α* as a reference. Euclidean distance was the distance measure used for HCA. *Red* gene names are hypothetical PC biosynthesis genes which may form the ω-oxo acid traumatin. *Green* gene names are associated with PC biosynthesis. *Blue* gene names are associated with JA biosynthesis. *Dashed arrow lines* indicate hypothetical origin of PC acyl CoA starter units. Transcript abundance was expressed as 40-ΔCq, a log2 measure of the transcript amount from the target gene to the reference gene (see Methods); *AAE*, acyl-activating enzyme; ALDH, aldehyde dehydrogenase (EC 1.2.1.3); *ALDH*, aldehyde dehydrogenases (EC 1.2.1.3); *AOC*,allene oxide cyclase (EC:5.3.99.6); *AOS*, allene oxide synthase (EC 4.2.1.92); *2-AR*, 2-alkynal reductase; *GPPS*, geranyl-diphosphate synthase; *HI*, (3*Z*):(2*E*)-hexenal isomerase; *HPL*,, hydroperoxide lyase (EC:4.1.2.-); 13*S*-HPOD, 13(*S*)-13-hydroperoxy-9(*Z*),11(*E*)-octadecadienoic acid; 13*S*-HPOT, 13(*S*)-hydroperoxy-9(*Z*),11(*E*),15(*Z*), octadecatrienoic acid; *JMT*, jasmonic acid carboxyl methyltransferase (EC 2.1.1.141); *LOX*, linolenate 13*S*-lipoxygenase; *LOX-L*, linoleate 13*S*-lipoxygenase 2 like; *OAC* - olivetolic acid cyclase (EC:4.4.1.26); OPR3, oxophytodienoate-reductase 3 (EC 1.3.1.42); *PT1*, geranylpyrophosphate:olivetolate geranyltransferase (EC 2.5.1.102); *TKS*, tetraketide synthase (EC:2.3.1.206); Δ9-THCA, Δ9 tetrahydrocannabinolic acid; *THCAS*, Δ9 tetrahydrocannabinolic acid synthase (EC:1.21.3.7).

Metabolites which display a high degree of connectivity in interaction networks are considered to be biologically important (Pei et al., 2017). Among the centrally located intramodular hub metabolites and in the top 10 most connected metabolites in the turquoise module was a putative ω-oxo acid 12-oxo-(10*E*)-dodecenoic acid, commonly known as traumatin (**Figure 5D**; **Supplementary Table S6**). This metabolite had a MM of 0.99 (*p*-value 7.57E-14) in the turquoise module and is also in the top 10 most significantly correlated compounds to PC content in this module (*r* 0.99, *p*-value 2.05E-11) (orange bold in **Supplementary Table S6**). Analysis of the MS spectra of this peak at 7.715 min supported its annotation as traumatin (orange bold in **Supplementary Figure S3**). Traumatin is a product of lipoxygenase (LOX) and hydroperoxide lyase (HPL) activity, which, if cleaved from a C_18_ unsaturated FA, would result in the formation of C_6_ aldehydes that are hypothesized to be the progenitors of hexanoyl-CoA in PC biosynthesis (**Figure 6A**; **Supplementary Figure S1**). Interestingly, we also identified other putative ω-oxo-acids and structurally related FAs in the turquoise module, including a C_12_ ω-oxo-acid (5*Z*,8*Z*,10*E*)-12-oxo-5,8,10-dodecatrienoic acid (*r* 0.98, *p*-value 2.77E-11) and a C_10_ oxo-acid 9-oxo-decenoic acid (*r* 0.98, *p*-value 1.07E-10) which both had a similar level of MM and correlation to PC content (orange bold in **Supplementary Tables S6**, **S7** and **Supplementary Figures S4**, **S5**), indicating that multiple putative LOX-HPL cleavage products have a strong relationship with PC content. We also searched for intermediates associated with other metabolic routes capable of producing short-medium chain acyl-CoA PC precursors, including those associated with branched-chain amino acid catabolism & α-ketoacid elongation (e.g., 2-oxo-valeric, 2-oxo-hexanoic acid and 2-oxo-heptanoic acid (Kroumova et al., 1994) (scenario 1 **Supplementary Figure S1**). However, these metabolites were not found among the modules (**Supplementary Tables S6**, **S7**), and we did not detect any compounds that matched the molecular formula and mass of these intermediates from the MS data (data not shown).

### 3.7 PC and MeJA biosynthesis gene expression

To further investigate the origin of oxylipin-derived FA starter units and their potential role in PC metabolism (**Figure 6A**), qPCR analysis was performed on two candidate oxylipin-related genes hypothesized to contribute to the production of hexanoyl-CoA in *C. sativa* plants (**Figures 6B, C**). These genes, which encode a linoleate 13*S*-lipoxygenase-like (LOC115719612; *LOX-L*) and a hydroperoxide lyase (LOC115698766; *HPL*), have previously been shown to have a high level of expression within isolated *C. sativa* glandular trichomes (Livingston et al., 2020). Consistent with these observations, *LOX-L* and *HPL* showed a higher level of relative transcript abundance in the inflorescence as compared with other JA and oxylipin genes (*LOX*, *AOC, OPR3, AOS* & *JMT*) (**Figure 6B**). *LOX-L* also had higher levels of transcript abundance in the inflorescence under control and treated groups compared with the JA-associated *C. sativa* gene *LOX* (LOC115707105), which encodes a protein with high homology to linolenate 13*S*-LOX (*At*LOX3, 96% query cover, 74.4% identity) (**Figure 6B**). Moreover, cluster analysis showed that the inflorescence transcript response to changes in MeJA and neomycin treatments for *LOX-L* and *HPL* were similar to other known PC biosynthesis genes and *LOX-L* clustered closely with *THCAS* (**Figure 6B**). Therefore, there is congruence of *LOX-L* and *HPL* expression patterns with both the previously published analyses using isolated trichomes.

Interestingly, the *LOX-L* and *HPL* expression patterns were similar in both the inflorescence and leaf samples (compare **Figures 6B, C**). This contrasts with the known PC genes *OAC*, *PT1* and *THCAS* which showed much lower levels of expression in the leaf, consistent with their function in PC biosynthesis within inflorescence-related tissues where capitate stalked trichomes are in highest abundance (**Figure 6C**). *JMT*, which is known to respond to MeJA treatment (Seo et al., 2001), showed the highest level of responsiveness to the MeJA treatment compared with the other PC and JA genes in both tissue types (**Figures 6B, C**). Despite *GPPS* being important for the synthesis of PC isoprenoid precursors (**Figure 6A**), this gene also showed a high level of expression in both tissues which may be a result of its distal role in PC synthesis compared with *OAC*, *PT1* and *THCAS* (**Figures 6B, C**). The ubiquitous expression patterns of *LOX-L* and *HPL* paralogs in the leaf and inflorescence suggests that they are constitutive genes and have other functions unrelated to PC metabolism.

## 4 Discussion

### 4.1 Effects of MeJA on plant morphology and metabolite content

Jasmonates, including MeJA, are important regulators of plant growth and development, with reductions in plant height and biomass following MeJA treatment having been reported across a number of diverse plant lineages, including sunflower (*Helianthus annuus*), tomato (*Solanum lycopersicum*), and soybean (*Glycine max*) (Boughton et al., 2005; Tian et al., 2012; Salari and Mansori, 2013; Li et al., 2018). While the mechanisms underlying JA-mediated plant growth inhibition are not well characterized, changes in cell proliferation, photosynthetic activity and accelerated senescence have been reported (Maserti et al., 2011; Shan et al., 2011; Noir et al., 2013). Concordant with the metabolomic analysis of *Arabidopsis* wild type and JA-deficiency mutant *opr3* under MeJA treatment (Cao et al., 2016), nitrogen and amino acid metabolism pathways relating to glutamine and proline metabolism identified in the blue modules were negatively impacted following JA treatment in *C. sativa* (**Table 1** and **Figures 4B**, **5A**). As previously suggested, changes in nitrogen assimilation and amino acid synthesis may be contributing to the reduction in plant growth (Cao et al., 2016). A dwarfing phenotype is highly desirable in medicinal cannabis production systems, as the contraction of internodes can result in the formation of compact inflorescences with a high harvest index (high ratio of floral tissue to shoot weight), and this has the potential to improve inflorescence or ‘bud’ potency *via* the enrichment of perigonal bracts, which support a high density of capitate stalked trichomes (Small, 2018). Given the reductions in height, extension of side branches and stem biomass in the MeJA-treated plants (**Figure 1**), this phenotypic response should be examined using a larger cohort of *C. sativa* phenotypes to quantify the impact of MeJA treatment on inflorescence structure and fine-branching architecture.

Previous analyses on the impact of MeJA on whole plant PC content and composition have been inconclusive. Increases in Δ9-THC content along with decreases in CBD content were reported in the leaves of plants treated with 1 and 5 µM MeJA, while treatments at 10 and 100 µM MeJA suppressed both PCs when compared with the controls (Salari and Mansori, 2013). Single applications of 0.1 – 1 mM MeJA to two-week-old medicinal cannabis flowers increased Δ9-THC content. However, this effect was transient over the four-week observation period following MeJA exposure, and in week 4, PC content was only marginally improved by ~5% when compared with the 0 mM MeJA control (Apicella et al., 2022). The phenotypic responsiveness to MeJA treatment varies between species and is dependent on the concentration and frequency of application (Li et al., 2018; Fedderwitz et al., 2020). As such, it is common practice to apply a wide range of concentrations that exceed 1 mM to determine the optimal dosage (Boughton et al., 2005; Li et al., 2018). Our observations of a dose-dependent increase in PC content using a broader molar range shows unambiguously that MeJA affects PC content (**Figure 2B**). We purposely targeted the early to mid-flowering stages prior to peak PC concentration to study the responsiveness of *C. sativa* floral tissues to MeJA treatment (De Backer et al., 2012). To fully understand the impact of MeJA on PC yield, this work could be extended to later flowering stages with consideration of other major PC chemotypes as well as medicinal cannabis subtaxa which accumulate higher levels of PCs (De Backer et al., 2012; Lynch et al., 2016). We included a negative control, the polycationic aminoglycoside antibiotic neomycin, which, among other responses, has been shown to inhibit cytosolic Ca^2+^ concentration, protease activity, terpenoid accumulation and *LOX* expression inducible by jasmonates in both mono- and di-cotyledonous plants (Vadassery et al., 2014; Vadassery et al., 2019). The metabolomes of neomycin-treated plants did not deviate significantly from the 0 mM MeJA control plants and so examination of neomycin at higher molar concentrations or the use of other jasmonate inhibitors, such as jarin-1, should be explored (Meesters et al., 2014; Vadassery et al., 2014). While our own observations do not support any obvious increases in glandular (either sessile or capitate) trichomes on the leaves and floral tissues of *C. sativa* that would lead to a doubling of PC content, increases in trichome density following MeJA treatment have been widely reported in plants (Boughton et al., 2005; Tian et al., 2012; Li et al., 2018). To understand the impact of MeJA on trichome number and morphology, comprehensive characterization of trichome populations throughout anthesis should be performed.

### 4.2 Origins of PC polyketide starter units

Despite the potential for interaction between oxylipin and PC biosynthesis, few studies have evaluated the impact of the oxylipin phytohormone MeJA on PC biosynthesis. As is evident from the untargeted metabolomic analysis of inflorescences and changes in gene expression observed in this study (**Figures 4A, B**, **6B**), the effects of MeJA treatment on PC content are unlikely to be restricted to PC precursor availability. However, given that PC content did increase following MeJA application, it seems likely that the synthesis of precursor compounds would closely follow that of the end products. Comparison of the methanol-soluble metabolomes of control and MeJA treated plants indicated a strong positive correlation of oxylipin metabolites with PC content (**Figure 5C** and **Supplementary Tables S5**, **S6**). Of these, the hub metabolite ω-oxo acid annotated as traumatin was one the most highly connected metabolites within the eigenmetabolite turquoise module and one of the most highly correlated to PC content.

Traumatin is one of two products formed *via* the wound-inducible cytochrome P450 enzyme HPL (Kallenbach et al., 2011). 13*S*-LOXs initiate this oxylipin biosynthetic reaction in the plastid by di-oxygenating C_18_ PUFAs, such linoleic and α-linolenic acid, with HPL cleaving dienoic (13*S*-HPOD) and trienoic (13*S*-HPOT) acids, respectively (**Figure 6A**). Cleavage of linoleic and α-linolenic derived hydroperoxy products will form the C_12_ FA, 12-oxo-(9Z)-dodecenoic acid, which is chemically isomerized to 12-oxo-(10*E*)-dodecenoic acid (traumatin). This chloroplastic LOX-HPL pathway also produces C_6_ aldehydes from either linoleic or α-linolenic acid C_18_ PUFA (**Figure 6A**). Cleavage of the hydroperoxy product 13*S*-HPOD releases hexanal, and this would require enzymatic conversion from an aldehyde to FA by an as yet unidentified short-chain aliphatic alcohol dehydrogenase or aldehyde dehydrogenase (ALDH) (Končitíková et al., 2015). For the 13*S*-HPOT derivative (3*Z* )-hexenal, two additional chemical or enzymatic steps would be required, potentially involving a cytosolic (3*Z*):(2*E*)-hexenal isomerase and 2-alkenal reductase (2-AR) (Kunishima et al., 2016). These C_6_ aldehydes, also known as green leafy volatiles (GLV), release a so-called ‘green odor’ like fragrance (Howe et al., 2000; Grechkin, 2002). While these C_6_ aldehydes weren’t identified in the metabolite datasets, the detection of traumatin is consistent with these being present and therefore a means by which hexanoic acid levels can be increased leading to higher OA levels for PC biosynthesis. Moreover, increases in both hexanal and hexenal have been reported in barley leaves following MeJA treatment (Kohlmann et al., 1999).

The involvement of the LOX-HPL pathway in PC biosynthesis was originally postulated from the analysis of trichome expressed sequence tags, with a high representation of transcripts encoding desaturases, LOXs and a HPL supporting this biosynthetic origin (Marks et al., 2009; Stout et al., 2012). More recently, RNA-Seq analysis of isolated trichomes shows strong transcriptional correlation of *LOX-L* (LOC115719612), *HPL* (LOC115698766) and the cytosolic hexanoyl-CoA synthetase with the CBD(A) determining gene *CBDAS* (Livingston et al., 2020), while proteins corresponding to these specific *LOX-L* and *HPL* paralogs are also present in the proteome of capitate stalked trichome heads containing the secretory cells (Conneely et al., 2021). In our analysis, the predicted linoleate *LOX-L* and *HPL* genes were highly expressed in the inflorescences of control and MeJA treated plants, and their expression patterns closely aligned with the known PC biosynthesis gene *THCAS*, but these only showed muted responses to MeJA when compared with *JMT* (**Figure 6B**). Previously, exogenous MeJA has been shown to increase *JMT* expression (Seo et al., 2001), and this was apparent in this study with qPCR analysis of the leaves and inflorescences treated with high concentrations of MeJA (**Figure 6B**). The expression patterns we observed for *HPL* are also analogous to previous experiments, with *HPL* having high levels of expression in the tomato leaf and flowers and being non-inducible by exogenous MeJA in *Arabidopsis* (Bate et al., 1998; Howe et al., 2000). Time course analysis of tomato leaves also shows differential activation of *LOX* paralogs under MeJA treatment, with expression patterns being transient in the 24 h post MeJA exposure (Upadhyay and Mattoo, 2018). Another possibility for the low responsiveness of *HPL* and *LOX-L* to MeJA is that upstream genes inducible by MeJA are driving changes in precursor availability, such as those encoding FA desaturases that facilitate the conversion of the monounsaturated oleic acid to linoleic and α-linolenic acid (Xue et al., 2017). Transcriptomic analysis of *C. sativa* plants under MeJA treatment may delineate the genetic determinates contributing to PC precursor biosynthesis.

Both C_3_ and C_5_ alkyl PCs were significantly correlated with traumatin-related compounds, possibly indicating that the enzymatic machinery for alkyl side chain length is shared among chemotypes. While alkyl side chain segregation patterns deviate from Mendelian expectations, digenic or oligogenic modes of inheritance are predicted for the C_3_ alkyl side chain chemotype (De Meijer and Hammond, 2016; Welling et al., 2019), suggesting that a limited number of genes/proteins are contributing to alkyl side chain analogue composition. While we are unaware of reports of a 13*S*-LOX that has activity towards C_16_ PUFAs, such a reaction has the capacity to form traumatin and a C_4_ aldehyde following HPL activity. 13*S*-LOXs are capable of synthesizing C_5_ aldehydes in what is believed to be a HPL-independent mechanism *via* a β-scission reaction (Shen et al., 2014). Recent genome-wide analysis shows 21 lipoxygenases in *C. sativa*, including a large cluster of 13*S*-*LOX* paralogs on chromosome 2, which includes *LOX-L* (Borrego et al., 2022). C_16_ PUFAs, such as hexadecatrienoic acid, can also serve as substrates in the LOX-HPL pathway, yielding a C_10_ ω-oxo acid 10-oxo-7-hydroxy-(*E*)-8-decenoic acid in replacement of traumatin (Nakashima et al., 2013). The presence of multiple putatively annotated and structurally related C_12_ and C_10_ oxo acids in the turquoise module with a high correlation to PC content (*r* 0.98) may potentially be reflective of the flexibility of oxylipin biosynthesis in *C. sativa* inflorescences.

The analysis of a mixed chemotype capable of producing high levels of both C_3_ and C_5_ alkyl PC showed changes in the alkyl side chain PC ratio that favored the production of the C_3_ alkyl analogues in the high MeJA treated plants. At the time of the analysis, the certified reference standard for the C_3_ alkyl side chain analogue of CBGA, cannabigerovarinic acid (CBGVA), was commercially unavailable which prevented quantitative analysis and comparison of these PC pathway intermediates. We also searched the MS data for compounds which matched the molecular formula and mass of the PC starter units hexanoyl-CoA and butanoyl-CoA, but these were not detected. While hexanoyl-CoA has been identified in the female flowers of *C. sativa*, the values reported were extremely low in the 10-20 pmoles g^-1^ FW range when using a multiple reaction monitoring (MRM) method by UPLC-MS/MS (Stout et al., 2012). These methods typically require specialized sample preparation (e.g., deproteinization by halogenation and purification by solid phase extraction (Jones et al., 2021)) which may also explain why these compounds were not observed from our analysis of methanol-soluble metabolomes. The development of targeted and highly sensitive mass spectrometry methodologies for analysis of short to medium chain acyl-CoAs in the PC-producing glandular trichomes could improve understanding of alkyl side chain analogue partitioning under MeJA application.

## 5 Conclusion

Using targeted metabolite profiling we showed that PC content increased with MeJA concentration in a dose-dependent manner, with approximately two-fold increase in PC levels at the highest MeJA treatment. While efforts were made to select a genotype capable of producing PCs with divergent alkyl side chain lengths and that was representative of the chemical diversity observed within the broader *C. sativa* gene pool, this analysis should also be extended to genotypes which exhibit other unusual PC profiles, such as those that produce either only CBGA or very low amounts of PCs (De Meijer and Hammond, 2016), to determine if MeJA associated changes are consistent across different chemical phenotypes and genetic backgrounds. Our untargeted analysis of the metabolomes of control and MeJA-treated plants also contributes to the growing body of literature on the importance of the oxylipin pathway in regulating specialized metabolism with insights beyond model plant species to the economically important plant *C. sativa*. We found that a number of metabolites within the oxylipin pathway are strongly correlated with PC content. While the putative identification of traumatin, a cleavage product of the oxylipin LOX -HPL pathway hypothesized to form PC precursors, may potentially suggest interaction between these pathways, these findings are correlative and therefore may not imply causation. To deepen our understanding of the relationship between oxylipin and PC biosynthesis pathways, gene silencing of *LOX-L* and *HPL* combined with ± MeJA treatments as well as biochemical assessment of recombinant LOX-HPL cleavage products would contribute to elucidating the origin of PC starter units. Irrespective of PC precursor synthesis, the metabolomic analysis presented here provides a strong platform to accelerate understanding on the role of oxylipin plant hormones on PC production and metabolism more broadly. Harnessing these interactions and resolving the molecular mechanisms underlying changes in specialized metabolism has the potential to provide new avenues for the biotechnological enhancement of *C. sativa*. Ultimately, this could also result in the development of novel chemotypically elite genotypes for medicinal as well as industrial end-uses.

## Data availability statement

The original contributions presented in the study are included in the article/**Supplementary Material**. Further inquiries can be directed to the corresponding author.

## Author contributions

MW wrote the manuscript and performed the metabolite analysis. MW, MO’B, and MSD designed the experiments. MW grew the plants and assisted MO’B with the phytohormone treatments. JC performed the qPCR analysis and assisted with the metabolite analysis. MAD developed the analytical method, performed the data acquisition, and assisted with the metabolite analysis. MSD and AB provided substantial contributions to conception and design of the research project and performed detailed review and revision of the manuscript. All authors contributed to the article and approved the submitted version.
