# Supplementary material for: Metabolomic analysis of methyl jasmonate treatment on phytocannabinoid production in Cannabis sativa
Source: Front Plant Sci. 2023 Mar 21;14:1110144. doi: 10.3389/fpls.2023.1110144 (PMC10070988; doi:10.3389/fpls.2023.1110144)
Supplement: Supplementary file 1 [file DataSheet_1.docx]

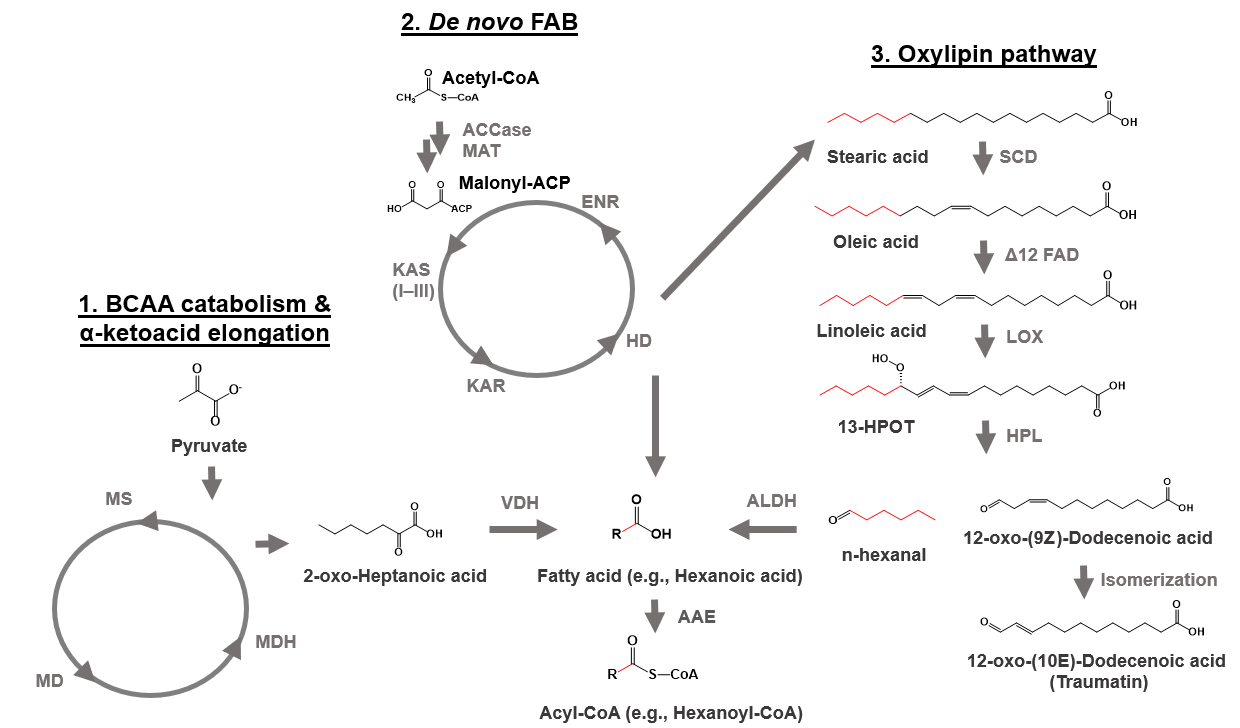


**Supplementary Figure S1**. **Schematic overview of putative biochemical pathways leading to the PC acyl-CoA polyketide starter units.** AAE - acyl-activating enzyme; ACCase - acetyl-CoA carboxylase (EC 6.4.1.2); ALDH - aldehyde dehydrogenase (EC 1.2.1.3); BCAA - branched-chain amino acid; ENR - enoyl-ACP reductase; FAB – fatty acid biosynthesis; FAD - fatty acid desaturase; KAS - β-ketoacyl-ACP synthase; KAR - 3-oxoacyl-[acyl-carrier-protein] reductase (EC 1.1.1.100); HD - 3-hydroxyacyl-ACP dehydratase; HPL - 1-Hydroxy-2-methyl-2-butenyl 4-diphosphate reductase (EC:1.17.7.4); MAT - malonyl-CoA: acyl carrier protein malonyltransferase (EC 2.3.1.39); MD - 3-isopropylmalate dehydratase (EC 4.2.1.33); MDH - 3-isopropylmalate dehydrogenase (EC 1.1.1.85); MS - 2-isopropylmalate synthase (EC 4.1.3.12); LOX -13S-lipoxygenase; SCD - stearoyl-CoA desaturase (Delta-9 desaturase) (EC:1.14.19.1); VDH - 2-oxoisovalerate dehydrogenase (EC 1.2.1.25)

**
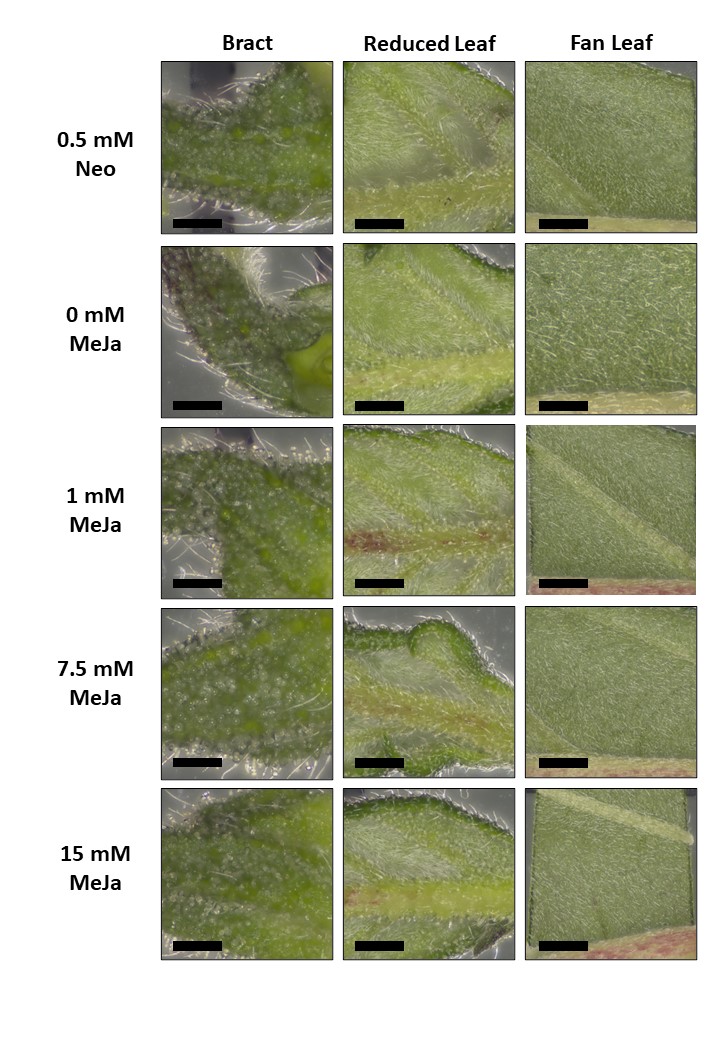
**

**Supplementary Figure S2**. **Foliage (fan leaves) and floral (perigonal bracts and reduced leaves) plant tissues/organs treated with methyl jasmonate (MeJA) and neomycin.** Images show the basal position of the leaves and were taken 14 d following the initial MeJA treatment and 24 h after the third and final whole-plant foliage application. Leaves were removed from the subapical phytomere of plant clones. *Black scale bar* = 1 mm.


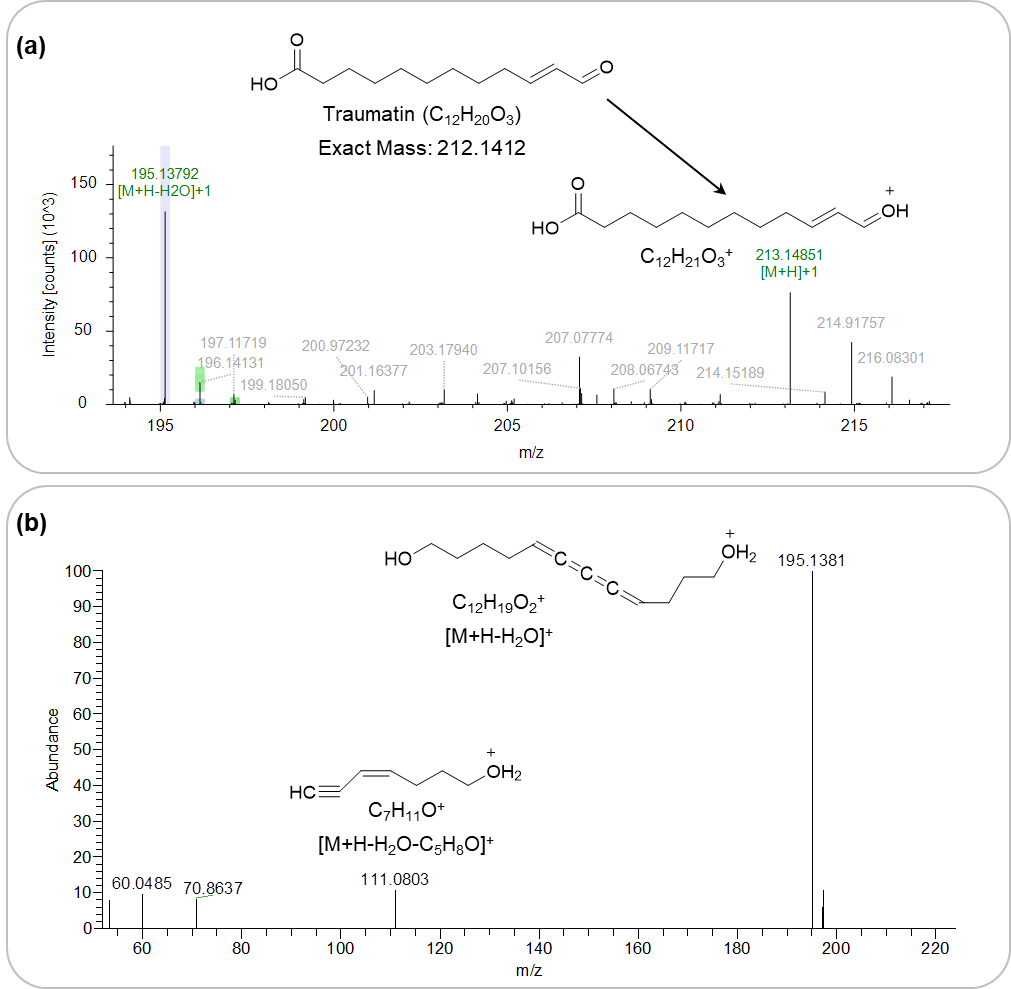


**Supplementary Figure S3**. **Mass spectra in positive mode of the peak at 7.71 min, which was annotated as traumatin.** **(a)** Structure of traumatin and the [M+H]^+^ ion with *m/z* 213.1485; and **(b)** observed MS^2^ spectrum of the extracted ion (*m/z* 213.1485). The most intense fragment ion was *m/z* 195.1381, which corresponded to a loss of water [M+H-H_2_0]^+^. The other fragment ion at *m/z* 111.0803 has a predicted composition of C_7_H_11_O^+^, most likely arising from the cleavage of the oxy-pentyl end of the [M+H]^+^ ion at the olefinic bond. Fragment Ion Search (FISh) is an algorithmic measurement of the percentage coverage of MS^2^ ions which match *in silico* fragmentation patterns from the HighChem™ Fragmentation Library™. The FISh coverage score of 100 percent indicated support of the chemical structure being traumatin, with Fragment ions matching the predicted MS spectrum for traumatin in The Human Metabolome Database (<https://hmdb.ca/spectra/ms_ms/67374>).

Reference: Allen F, Greiner R, Wishart D: Competitive fragmentation modeling of ESI-MS/MS spectra for putative metabolite identification. Metabolomics. 2015 11(1):98–110. doi: 10.1007/s11306-014-0676-4.


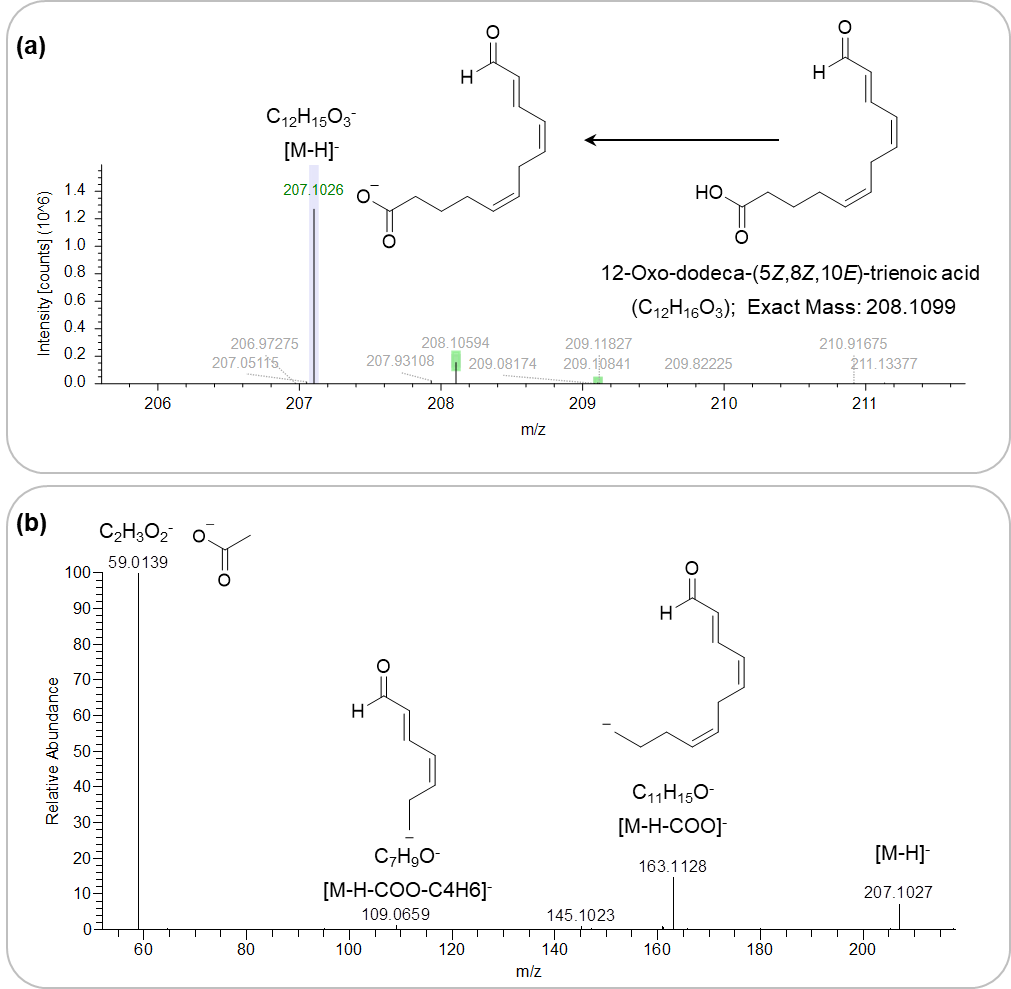


**Supplementary Figure S4.** **Mass spectra in negative mode of the peak at 8.21 min, which was annotated as 12-oxo-dodeca-(5Z,8Z,10E)-trienoic acid.** **(a)** Chemical structure and the [M-H]^-^ ion with *m/z* 207.1026; and **(b)** MS^2^ fragment ions in negative mode. The most intense MS^2^ fragment ion observed in negative mode was *m/z* 59.0139 that corresponded to the cleavage of the terminal carboxyl group. The other fragment ion *m/z* 163.1128 corresponded to the loss of the carboxyl group and *m/z* 109.0659 to the subsequent loss of a butyl group. Fragment Ion Search (FISh) was 71.43%.

**
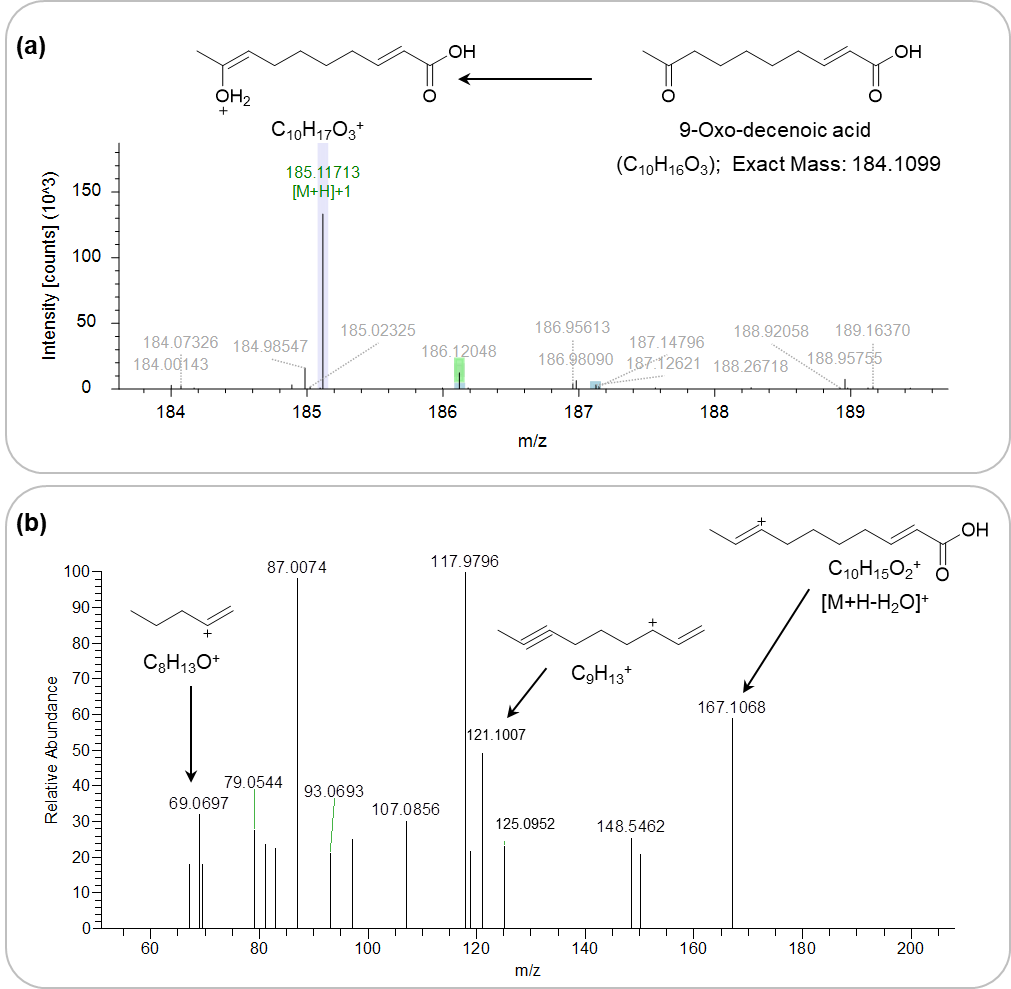
**

**Supplementary Figure S5.** **Mass spectra in positive mode of the peak at 13.68 min, which was annotated as 9-oxo-decenoic acid.** (a) Chemical structure and MS spectrum with [M+H]^+^ at *m/z/* 185.1171; and (b) observed MS^2^ fragment ions. The peak was annotated as 9-oxo-decenoic acid, and the Fragment Ion Search (FISh) was 60 percent coverage with only three fragment ions matched to the predicted composition. There are other components present in this peak and the extracted ion for this compound indicated a relatively low amount in the extract matrix.
